# Beyond GPT-4: The Rapidly Evolving Potential of Large Language Models for Clinical Guideline Improvement

**DOI:** 10.2196/95004

**Published:** 2026-04-10

**Authors:** Scott D Nelson, Adam Wright

**Affiliations:** 1 Department of Biomedical Informatics, School of Medicine, Vanderbilt University Medical Center, 3401 West End Ave, Nashville, TN, 37203, United States, 1 6158759347

**Keywords:** artificial intelligence, clinical guidelines, large language model, patient safety, readability, clinical decision support

## Abstract

This commentary reviews the study by Jones et al, which evaluated whether GPT-4 could improve the readability of injectable medication guidelines while preserving important safety information. The study found that GPT-4 produced modest readability gains comparable to manual revision, but also introduced omissions and meaning changes in a minority of sections. These findings highlight both the potential and limitations of early large language models (LLMs) in clinical contexts. However, this study reflects the capabilities of a specific model in a rapidly evolving domain. Since the release of GPT-4, advances in multistep reasoning, model-critique workflows, and structured validation have substantially improved the ability of newer systems to detect omissions, maintain factual fidelity, and support controlled editing. As a result, some documented limitations may stem from the constraints of a single-model, single-pass workflow rather than intrinsic flaws in LLM-assisted guideline revision. This commentary highlights the need for evaluation frameworks that can keep pace with LLM progress and emphasizes that clinical oversight and user-centered testing remain essential. Updated research using contemporary models is needed to determine how emerging architectures can more safely support clarity, consistency, and maintenance of clinical guidelines.

## Introduction

In their recent study, Jones et al [1] evaluated a GPT-4–based pipeline for improving the readability of 20 guidelines from the United Kingdom’s National Health Service Injectable Medicines Guide (IMG). The authors found that GPT-4 produced modest but statistically significant readability improvements, and expert pharmacist reviewers rated the revised versions as easier to understand for 26 of 60 (43%) of the ratings. The gains in readability were comparable to those achieved by manual revisions by guideline authors; however, the greatest readability improvements were for two guidelines (aminophylline and voriconazole) using iterative user testing from a previous study.

Notably, using the large language model (LLM) was not without risk. At least one pharmacist reviewer identified omissions in 30 of 153 subsections (20%), additions in 7 subsections (5%), and changes in meaning in 18 subsections (12%). Eight subsections had omissions identified by all 3 reviewers, but no additions or changes in meaning were unanimously flagged. Overall, 65% of all identified issues were flagged by only a single reviewer. The authors concluded that GPT-4 could help augment, rather than replace, manual expert review and user-centered testing to improve guideline readability.

## The Challenge of Evaluating a Moving Target

Interpreting these findings requires an appreciation for how rapidly LLM technology has advanced since the release of GPT-4 in March 2023, an extraordinarily long time ago in the current field of artificial intelligence advancements. By the time the study was published, newer models had already introduced major improvements in error checking, multistep reasoning, and structured critique workflows [2,3]. This creates a temporal mismatch where clinical research and guideline development cycles operate on the scale of *years*, while LLM development cycles operate on the scale of *months* and could continue to accelerate [4]. Thus, this study should be viewed as an evaluation of a specific model at a fixed point in time, not a judgment on the overall potential of LLMs in guideline development and review workflows. This is not a criticism of the study itself, as the authors designed a careful, well-controlled evaluation and their findings are valuable precisely because they document specific failure modes that future systems must address. Rather, it is a call to develop more agile evaluation frameworks that can keep pace with technological change, so that evidence generation does not perpetually lag behind the tools available for deployment.

For example, the GPT-4 pipeline relied on a single model for both the editing and quality assurance steps. This architecture creates an inherent tension, since simplification commonly results in removing content, so omissions could be somewhat expected. Newer multiagent systems separate generation from critique, allowing an editor model to propose revisions while a critic model checks for completeness, consistency, and factual accuracy [5]. Structured reasoning frameworks, such as tree-of-thought and self-consistency, enable models to justify edits and cross-check them before finalizing. Skill-based architectures allow explicit function calls to validate medication names, units, values, and section completeness, replacing soft prompts with enforceable programmatic safeguards, while dynamic prompt optimization can iteratively refine instructions to prevent prompt-induced errors. These advances do not eliminate the need for clinician oversight, but they offer more robust mechanisms for preserving informational fidelity while improving readability.

## Contextualizing the Use Case

The IMG guidelines represent a relatively favorable use case for LLM revision: procedural, deterministic instructions with clear ground truth. This contrasts with other clinical practice guidelines, such as those for disease management, which must synthesize heterogeneous evidence, navigate gaps, and rely on expert consensus across nonrepresentative study populations, posing far greater challenges. In this example, the study evaluated GPT-4’s performance on the IMG, which is a nationally curated, professionally edited resource. Improving readability on an already well-crafted guideline is inherently challenging, yet the model sill produced modest readability improvements. In practice, LLMs may offer even greater value for locally developed clinical guidelines and documents, which are often produced under time pressure with less editorial rigor. Enhancing the clarity and consistency of these documents could improve staff comprehension and ultimately lower the risk of downstream errors. This study tested the LLM against the hardest version of the problem, improving something already well-crafted, while the real-world opportunity may lie in lifting up the documents that need it most.

## The Continued Need for User-Centered Testing

User testing remains the guideline improvement technique with the strongest evidence base, and the authors’ own data confirm this. Future studies should also include the intended end users. For example, the IMG is primarily used by nurses, who may prioritize quick scanability and visual hierarchy over the pharmacological completeness that pharmacist reviewers would naturally emphasize. The interaction between content accuracy and practical usability can only be fully understood by the people who use these documents at the point of care.

## Beyond Readability

Improving readability is only one part of making guidelines more usable. Simplifying text naturally risks omitting information, while providing excessive detail poses its own risks: dense guidelines can cause clinicians to overlook or misinterpret critical information. In the study, 65% of errors were identified by only a single pharmacist, suggesting that many issues were subtle and difficult to detect. The real question is how we can preserve and present essential information in the clearest, most usable form. Newer multimodal models offer approaches beyond text alone. They can generate diagrams, flowcharts, and annotated step-by-step visuals, which may communicate procedural information, such as reconstitution or infusion setup, more effectively than narrative text [6]. These multimodal formats can help reduce cognitive load and help clinicians understand complex instructions more intuitively.

Furthermore, LLMs have broader potential in the guideline ecosystem. They could support translation into other languages, improving access and equity in multilingual care settings [7], though clinical translation would require rigorous verification [8]. LLMs may also enable just-in-time guidance by retrieving and tailoring the relevant portion of a guideline to a clinician’s immediate question, which would often be more valuable than improving long documents clinicians may not have time to read. In addition, LLMs could assist with clinical decision support (CDS) maintenance, helping translate updated recommendations into structured CDS logic, or flag conflicts between new evidence and existing rules, reducing alert fatigue and easing the burden of keeping CDS systems current [9]. These applications warrant dedicated study using current-generation models.

## Conclusion

Jones et al [1] provide a rigorous, timely evaluation of GPT-4’s capabilities and limitations in revising medication guidelines. Their findings identify clear failure modes that future systems must overcome. As LLM architectures continue to advance, updated evaluations are essential to determine how well newer systems address the documented issues and how they can safely support clinicians, guideline authors, and health care organizations. None of these advances eliminate the need for clinician oversight or user-centered testing. The goal is to equip guideline authors and informatics teams with powerful tools for improving how clinical knowledge is communicated and delivered at the point of care. The evidence base must evolve alongside the technology.

## Abbreviations

CDS: clinical decision support
IMG: Injectable Medicines Guide
LLM: large language model
